# The hidden dangers of electronic cigarettes: e-cigarette, or vaping, product-use associated lung injury requiring extracorporeal membrane oxygenation

**DOI:** 10.36416/1806-3756/e20240163

**Published:** 2025-03-18

**Authors:** Anna Maria Garcia Cardoso, Wanessa Nayane Alves Rabelo, Stephan Adamour Soder, Fabíola Adélia Perin, Spencer Marcantonio Camargo

**Affiliations:** 1. Serviço de Cirurgia Torácica, Pavilhão Pereira Filho, Santa Casa de Misericórdia de Porto Alegre, Porto Alegre (RS) Brasil.

## TO THE EDITOR:

In recent decades, multiple efforts have been made to reduce population exposure to cigarettes, given that smoking is responsible for thousands of deaths annually. These actions have been effective, as evidenced by a reduction in the number of smokers worldwide in recent decades and by a slight reduction in the incidence of lung cancer.^1^ Conversely, a new form of nicotine exposure has become popular through electronic cigarettes, first commercially available in the early 2000s in China.^2^ Currently, the electronic cigarette industry generates $2.5 billion annually.^3^

Recent studies have shown that 4.9% of elementary school students and 20.8% of high school students in the United States report having used electronic cigarettes in the last 30 days. Between 2017 and 2018, there was a 78% increase in electronic cigarette use among American high school students.^3^ In Brazil, a national survey conducted in 2022 estimated that the prevalence of electronic cigarette use was 12.2%, with the predominant age group being 25-34 years.^4^ In southern Brazil, this rate is even more concerning, corresponding to 21.9% of the school-age population.^5^

Electronic cigarettes work by heating a liquid to create an aerosol that typically contains solvents such as propylene glycol and vegetable glycerin, as well as nicotine and flavorings; it can also contain chemicals such as tetrahydrocannabinol. Although the medium-term and long-term health effects of inhaling these substances into the lungs are still unknown, there have been numerous reports of patients who develop acute lung damage caused by inhaling the vapor produced by electronic cigarettes. E-cigarette, or vaping, product use-associated lung injury (EVALI) is a term that is used in order to describe lung injury caused by the use of electronic cigarettes. Symptoms include cough, dyspnea, chest pain, and hemoptysis. In severe cases, invasive ventilatory support such as mechanical ventilation and extracorporeal membrane oxygenation (ECMO) may be required; in extreme cases, lung transplantation may be required.^3^ ^,^ ^6^ ^,^ ^7^

Although there is no specific criterion defining electronic cigarette intoxication as a cause of lung injury, an algorithm was proposed by the U.S. Centers for Disease Control and Prevention along with the University of Rochester for the classification of acute lung injury caused by electronic cigarettes. Patients must present with cough and dyspnea, as well as systemic symptoms such as fatigue and fever. They should have a history of vaping in the last 90 days, as well as imaging showing diffuse bilateral infiltrates with ground-glass opacity, predominantly in the lung bases, with subpleural sparing. Infectious, neoplastic, cardiac, and rheumatologic causes should be excluded.^3^

Here, we report the first case of a patient in Brazil presenting with lung injury associated with electronic cigarette use and requiring ventilatory support and ECMO. Written informed consent was obtained from the patient for publication of this report and accompanying images. 

A 23-year-old male farmer presented with no history of allergies, surgery, or continuous medication use. The patient was obese (his BMI being 36 kg/m^2^) and a smoker, having smoked conventional cigarettes (40 cigarettes/day) since he was 14 years old and having replaced them with electronic cigarettes, smoked daily in the last 3 years. He reported no use of other drugs. 

The patient presented to the emergency department with cough, dyspnea, and fever, being prescribed amoxicillin and clavulanate, as well as symptomatic treatment. However, he developed persistent fever, cough, and worsening dyspnea, returning to the emergency department 48 h after initiation of treatment. The patient was hospitalized and started on ventilatory support via a Venturi mask, requiring intubation and mechanical ventilation. He was admitted to the ICU for 12 h. The patient was screened for respiratory viruses (influenza A, influenza B, SARS-CoV-2, and respiratory syncytial virus), but the results were negative. After five days of endotracheal intubation and mechanical ventilation, the patient presented with refractory hypoxemia (a PaO_2_/FiO_2_ ratio of 80) despite optimal ventilator management and pronation, with a peak pressure of 40 cmH_2_O. A decision was made to initiate ventilatory support with venovenous ECMO with two single-lumen cannulas (with blood being drained from the right femoral vein with a 23 Fr cannula and being reinfused into the right internal jugular vein with a 21 Fr cannula). On the same day, the patient was transferred to the ICU of the *Santa Casa de Misericórdia de Porto Alegre Pavilhão Pereira Filho*, located in the city of Porto Alegre, Brazil, for specialized care. 

The patient remained intubated for 12 days. He failed extubation, requiring reintubation within 48 h of planned extubation and remaining on mechanical ventilation for another 48 h and on ECMO for 14 days. 

Three BAL procedures were performed during his hospitalization, the BAL fluid samples being sent for bacteriological and mycological analysis. The first procedure was performed upon patient arrival; the second was performed on the following day; and the third was performed one week later. The samples from the third procedure showed bacterial growth (*Klebsiella pneumoniae*), and the patient was treated with antibiotics. 

**Figure 1:**
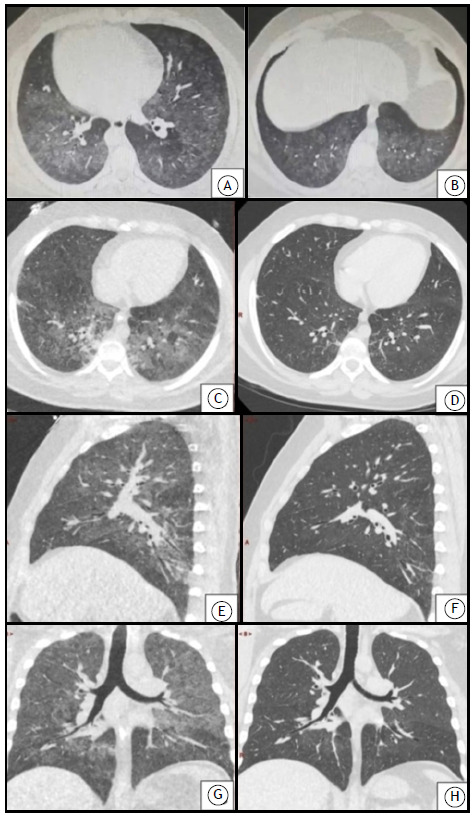
In A and B, axial CT scans of the chest of a male patient hospitalized with e-cigarette, or vaping, product use-associated lung injury. In C and D, axial CT scans of the lower lobes. In E and F, sagittal CT scans. In G and H, coronal CT scans. The scans on the left (E and G) were performed two days after extracorporeal membrane oxygenation removal, and the scans on the right (F and H) were performed two days before hospital discharge.

During his ICU stay, the patient was evaluated for autoimmune disease, being negative for antinuclear and anti-DNA antibodies. His ESR was = 6. Figure 1 shows chest CT scans performed at hospital admission, as well as comparative images taken a few days later. 

The patient was discharged after 26 days of hospitalization and is undergoing outpatient follow-up at this writing, showing progressive improvement. He has lost weight through dietary and physical activity adjustments, and is expected to return to work soon. 

The case reported here is important because of the increasing use of electronic cigarettes and the potentially harmful consequences of electronic cigarette use. Although electronic cigarettes have been prohibited by the Brazilian Health Regulatory Agency since 2009, the regulation of electronic cigarettes has been discussed by the Congress. This report adds to others describing cases of EVALI, serving as a warning to the medical community, public authorities, and the population at large about the risks associated with the use of electronic cigarettes. Although there have been reports of cases of EVALI requiring ECMO,^8^ ^,^ ^9^ this was the first such case in Brazil. 
